# Game Performance Evaluation in Male Goalball Players

**DOI:** 10.1515/hukin-2015-0090

**Published:** 2015-01-12

**Authors:** Bartosz Molik, Natalia Morgulec-Adamowicz, Andrzej Kosmol, Krzysztof Perkowski, Grzegorz Bednarczuk, Waldemar Skowroński, Miguel Angel Gomez, Krzysztof Koc, Izabela Rutkowska, Robert J Szyman

**Affiliations:** 1 The Józef Pilsudski University of Physical Education in Warsaw, Faculty of Rehabilitation, Poland; 2 The Józef Pilsudski University of Physical Education in Warsaw, Faculty of Physical Education, Poland; 3 Polytechnic University of Madrid, Faculty of Physical Activity and Sport Sciences, Madrid,, Spain; 4 Chicago State University, Faculty of Secondary Education, Professional Studies and Recreation, Chicago, Illinois, USA

**Keywords:** visual impairment, disability sport

## Abstract

Goalball is a Paralympic sport exclusively for athletes who are visually impaired and blind. The aims of this study were twofold: to describe game performance of elite male goalball players based upon the degree of visual impairment, and to determine if game performance was related to anthropometric characteristics of elite male goalball players. The study sample consisted of 44 male goalball athletes. A total of 38 games were recorded during the Summer Paralympic Games in London 2012. Observations were reported using the Game Efficiency Sheet for Goalball. Additional anthropometric measurements included body mass (kg), body height (cm), the arm span (cm) and length of the body in the defensive position (cm). The results differentiating both groups showed that the players with total blindness obtained higher means than the players with visual impairment for game indicators such as the sum of defense (p = 0.03) and the sum of good defense (p = 0.04). The players with visual impairment obtained higher results than those with total blindness for attack efficiency (p = 0.04), the sum of penalty defenses (p = 0.01), and fouls (p = 0.01). The study showed that athletes with blindness demonstrated higher game performance in defence. However, athletes with visual impairment presented higher efficiency in offensive actions. The analyses confirmed that body mass, body height, the arm span and length of the body in the defensive position did not differentiate players’ performance at the elite level.

## Introduction

Goalball is a Paralympic sport exclusively for athletes who are visually impaired or blind. The game is played by two teams of three players on a volleyball-sized court with a ball made of hard rubber with metal bells inside. The auditory cues made by bells inside the ball help to orient the players and indicate the direction of the oncoming ball (Davis, 2002). The purpose of the game is to score a goal by throwing the ball past the opponent’s goal line (the end line of the court), while the other team attempts to stop the rolled ball (IBSA, 2010). Although players are classified by their visual acuity into three classes – B1 (visual acuity poorer than LogMAR 2.60), B2 (visual acuity ranging from LogMAR 1.50 to 2.60 and/or visual field constricted to a diameter of less than 10 degrees) and B3 (visual acuity ranging from LogMAR 1.40 to 1 and/or visual field constricted to a diameter of less than 40 degrees), they all wear eyeshades during the goalball game (IBSA, 2010). Research on performance analysis in sport enhances the use of notational analysis in team sports to assess the performance of an individual, a team or elements of a team (McGarry et al., 2013). Performance analysis is based on performance indicators (also called performance variables) described as a selection or combination of action variables that aim to define some or all aspects of a performance and help to achieve sport success (Hughes and Bartlett, 2002). Notational analysis is focused on general match, tactical as well as technical indicators, and contributes to the understanding of the physiological, psychological, technical and tactical demands of sports disciplines.

Recently, some studies have attempted to assess game performance in such Paralympic sports as wheelchair basketball (Molik et al., 2009; Vanlandewijck et al., 1995; Vanlandewijck et al., 2003; Vanlandewijck et al., 2004), wheelchair rugby (Molik et al., 2008; Morgulec-Adamowicz et al., 2010; Sarro et al., 2010; Sporner et al., 2009) and ice sledge hockey (Kudláĉek et al., 2006; Molik et al., 2012). These studies have investigated performance differences during games between the classification levels of athletes with disabilities, and comparisons of teams’ performances relative to their ranking in competitions and players’ performances related to their game position. In goalball, research has been mainly focused on the intensity of effort during the game (Pilianidis et al., 2005), general physical fitness (Karakaya et al., 2009; Çolak et al., 2004), aerobic capacity (Gulick and Malone, 2011), postural stability (Aydoğ et al., 2005), anthropometrics (Calıskan et al., 2011), psychological strategies (Stamou et al., 2007) and throwing technique (Bowerman et al., 2011). No scientific evidence has been gathered using goalball game performance analysis.

Gualdi-Russo and Zaccagni (2001) stated that athlete’s anthropometry may represent important prerequisites for successful participation in any given sport. A few studies have examined the anthropometric profile of goalball players, but did not relate them to game performance (Karakaya et al., 2009; Calıskan et al., 2011). Therefore, an understanding of the basic anthropometric characteristics of elite goalball athletes may be important for accurate distribution of resources within a team. In goalball, length of the body in the defensive position, height and the arm span seem to be closely related to most goalball techniques and may influence performance.

The impact of the degree of visual impairment on the motor skill performance of individuals with visual impairment is still under discussion (Houwen et al., 2009; Juodžbalienė and Muckus, 2006). Although, goalball athletes wear blackened eyeshades during the game as an equalizer, it is important to examine the differences in game performance between players with total blindness (B1) and players with visual impairment (B2 and B3) as the degree of visual impairment may influence motor skill development.

The aims of the present study were (1) to describe game performance of elite male goalball players based upon the degree of visual impairment, and (2) to determine if game performance was related to anthropometric characteristics of elite male goalball players.

## Material and Methods

This study was approved and supported by the International Paralympic Committee (IPC) Sports Science Committee (SSC). Informed consent was required to participate as per ethics approval from the Ethics Committee.

### Participants

#### Game performance

A sample of 44 elite male goalball athletes participating in the Summer Paralympic Games in London 2012 and representing twelve top world teams (Algeria, Belgium, Brazil, Canada, China, Finland, Great Britain, Iran, Korea, Lithuania, Sweden and Turkey) took part in this study. To be included in the game performance analysis, an athlete had to participate for a minimum of 24 min totally and play in a minimum two games during the tournament. Athletes were categorized according to the degree of visual impairment into 2 groups: B1 (n = 15) and B2/B3 (n = 29).

#### Anthropometric measurements

From the 44 game performers who met the 24 min and two game criteria, 27 athletes representing eight teams (Algeria, Belgium, Brazil, Canada, Iran, Lithuania, Sweden and Turkey) volunteered to take part in anthropometric measurements. Those who chose not to participate indicated lack of time as the main reason for declining to take part in the research. Mean age of B1 players was 27.63, *s* = 5.16, and B2/B3 - 27.87, *s* = 8.16. Disability experience in years of B1 players was 21.36, *s* = 8.04, and B2/B3 - 26.12, *s* = 9.42. Training experience (years) of B1 players was 12.36, *s* = 6.12, and B2/B3 - 12.12, *s* = 7.16, and training frequency (hours per week) of B1 players was 12.63, *s* = 5.74, and B2/B3 - 9.25, *s* = 4.95. There were no significant differences between both groups in terms of the aforementioned data.

### Measures and Procedures

During training sessions prior to the tournament, 27 players representing eight teams completed a *Personal Questionnaire Form* (i.e., first and family name, age, country, type of disability, disability experience, training experience, and training frequency). Anthropometric measurements included body mass (kg), body height (cm), the arm span (cm) and length of the body in the defensive position (cm). Body mass was assessed using an AccuSway force platform (Advanced Mechanical Technology, Inc., Watertown, Massachusetts). Athletes were weighed in normal track clothing. Body height was measured in a standing position against a wall without shoes or socks using a headboard and carpenter’s tape vertically fixed to the wall. The arm span was measured in the same position, using carpenter’s tape horizontally fixed to the wall, as length from one end of an athlete’s arm (measured to the finger tips) to the other when raised parallel to the ground at shoulder height. Length of the body in the defensive position was measured using carpenter’s tape fixed to a floor, as the maximum horizontal distance from the wall to the farthest point of the finger tips, while the participant was lying on the floor on his preferred side with arms extended, legs and feet stretched as much as possible and toes touching the wall. The athlete was barefoot during this measurement. All 38 scheduled men’s games were recorded using video cameras placed on the opposite sides of the goalball courts. One camera recorded a wide plane of the matches from the centre line, while the second camera followed the ball during the matches. The position of each camera was negotiated with the IPC Sports Science Committee and the local organizing committee. During videotaping, the researchers noted the actual game situation (i.e., score of the game, a player’s uniform number on the court, referee’s decisions, and each player’s activeness as measured by the Game Efficiency Sheet for Goalball/GES-GB/ - see below). The games were analysed after the tournament by 8 independent observers. All observers were trained at special meetings held between September and November 2012. Finally, inter-observer and intra-observer reliability were established by statistical analysis (*r* > 0.93, *p* < 0.05, and *r* > 0.95, *p* < 0.05, respectively).

Each match was analyzed separately by two researchers (December 2012 – January 2013). All observations were recorded using the Game Efficiency Sheet for Goalball (GES-GB), developed by the authors. The following 23 game indicators were included for analysis: a good attack – a goal (AG), a missed attack – a shot of the opponent immediately after defense (AMS), a missed attack - defense and the ball out (AMDO), a missed attack – out (AMO), a missed attack - defense and lost contact with the ball (AMLC), a missed attack - defense and lost contact with the ball - ball back to the shooter (AMB), a good defense – the ball out (DGO), a good defense – a lost ball (DGL), a good defense – the defender keeps contact with the ball (DGC), a good defense – the second defender keeps contact with the ball (DGS), a good defense - lost contact with the ball – the ball back to the opponent (DGB), not a good defense (DN), a penalty good defense (PDG), a penalty not a good defense (PDN), a penalty attack good-goal (PAG), a penalty attack missed (PAM), a foul (FO), an attack spin a throw a good goal (APtG), an attack spin a throw missed (APtM), an attack run a good – goal (ARtG), an attack run missed (ARtM), an attack stand a good – goal (AStG), an attack stand missed (AStM). Additionally, the sum of attacks/shots (Asum), the sum of missed attacks/shots (AMsum), the sum of defenses (Dsum), the sum of good defenses (DGsum), the sum of penalty defenses (PDsum), the sum of penalty attacks (PAsum), the sum of attacks spin throw technique (APtsum), the sum of attacks run technique (ARtsum), and the sum of attacks stand (AStsum) were calculated. All indicators were calculated as average per game (24 min). The following formulae were developed for the efficiency indicators:

$$\begin{array}{l} \left(\text{Aef})\;\text{attack efficiency }[\%]=(\text{AG})*100/(\text{Asum}\right)\\ \left(\text{Def})\;\text{defense efficiency }[\%]=(\text{DGC})*100/(\text{Dsum}\right)\\ \left(\text{PDef})\;\text{penalty defense efficiency }[\%]=(\text{PDG})*100/(\text{PDsum}\right)\\ \left(\text{PAef})\;\text{penalty efficiency attack }[\%]=(\text{PAG})*100/(\text{PAsum}\right)\\ \left(\text{APtef})\;\text{attack spin throw efficiency }[\%]=(\text{APtG})*100/(\text{APtsum}\right)\\ \left(\text{ARtef})\;\text{attack run efficiency }[\%]=(\text{ARtG})*100/(\text{ARtsum}\right)\\ \left(\text{AStef})\;\text{attack stand efficiency }[\%]=(\text{AStG})*100/(\text{AStsum}\right) \end{array}$$

### Statistical Analysis

First, a descriptive analysis (means and standard deviations) of the data for general characteristics and anthropometric measures for each group was performed. Secondly, to verify the objectives of the study, a Mann-Whitney *U* test (non-parametric) was carried out in order to analyze the differences between athletes with total blindness (B1) and those with visual impairment (B2/B3) for all the game indicators studied. Thirdly, to analyze the impact of the following independent variables: body height (BH), the arm span (AS) and length of the body in the defensive position (LBDP) on the following dependent variables: (Aef) attack efficiency [%], (Def) defense efficiency [%], (PDef) penalty defense efficiency [%], (FO) foul, (APtef) attack spin throw efficiency [%], (ARtef) attack run efficiency [%], and (AStef) attack stand efficiency [%], seven linear regression models were applied. DV is the dependent variable (game indicators), *β_0_* is the intercept and *β_1_*, *β_2_* and *β_3_* indicate the impact of each predictor variable on the independent variables. Finally, *ɛ_i_* is the disturbance term. The model is as follows:

DV=β0+β1*BH+β2*AS+β3*LBDP+ɛi

Statistical analyses were performed using SPSS 18 software release for Windows. Statistical significance was set at *p* < 0.05.

## Results

Means and standard deviations for each game indicator by the degree of visual impairment are presented in Table 1. The results of the U-Mann Whitney test differentiating both groups showed that the players with total blindness obtained higher means than the players with visual impairment for the game indicators of the sum of defense (Dsum) (Z = −2.07; p =0.03), the sum of good defense (DGsum) (Z = −2.04; p = 0.04), and good defense - lost contact with the ball rebounding to the opponent (DGB) (Z = −2.60; p = 0.02). Conversely, the players with visual impairment obtained higher means than the players with total blindness for attack efficiency (Aef) (Z = −2.00; p = 0.04), the sum of penalty defenses (PDsum) (Z = −2.98; p = 0.01), penalty good defense (PDG) (Z = −2.18; p = 0.04), penalty not good defense (PDN) (Z = −2.22; p = 0.03), and fouls (FO) (Z = −3.10; p = 0.01).

Results for anthropometric measurements of goalball players (n = 27) with respect to the degree of visual impairment are presented in Table 2.

The effects of the three independent variables (anthropometric measures) on game performances are displayed in Table 3. The results showed that none of the seven linear regression models were statistically significant (p > 0.05) when predicting the effectiveness of each game indicator (attack efficiency, defense efficiency, penalty defense efficiency, the foul, attack spin throw efficiency, attack run efficiency, and attack stand efficiency). These results reflect that elite goalball players’ performances in game indicators are not directly affected by anthropometric variables.

## Discussion

The first aim of the present study was to describe game performance of elite male goalball players with regard to their degree of visual impairment. Comparisons of 39 different game performance variables showed significant differences between the athletes with total blindness (B1) and those with visual impairment (B2 & B3) in seven of the variables. B2 & B3 athletes achieved higher levels of effectiveness in attack (shooting). This higher efficiency could be advantageous for B2 & B3 athletes during the game against B1 athletes. As a consequence coaches may want to use athletes with visual impairment more frequently in offensive actions to achieve success.

Another finding was that players with visual impairment (B2 & B3) tended to commit a significantly higher number of personal penalties. As a result of the personal penalties, opponents scored by penalty shots. Most penalized errors were related to personal penalties (i.e. a short ball, a high ball, too many shots, too long time – more than 10 s - without shots). A personal penalty is a consequence of risky offensive actions. Personal penalties should be reduced to improve the competitive level of teams. The number of defensive penalties (the sum, good defense and not good defense actions) was higher among B1 players compared to B2 & B3 players.

This phenomenon is the result of a higher number of fouls by players with vision, as the player who fouls then defends against the penalty shot.

Players with total blindness defended more frequently than their teammates (the sum of defense) including blocking powerful shots that rebound back to the opponents. Those results could be due to the specialization of athletes with total blindness. It seems that coaches stress the role of athletes with total blindness in defense. Specific positioning during defense (defending wider range of the goal) can increase the number of opportunities that the players with total blindness have to defend the goal. However, it is also possible that the higher number of opportunities is a consequence of the opponent’s tactical solutions. It could be that the opponents try to shoot toward the so called weaker points of the opposite team i.e. athletes with total blindness. Moreover, shots on weaker defenders (athletes with total blindness) could reduce efficiency of power in offensive actions.

Analyses of game performance yielded some differences between goalball players with total blindness and those with visual impairment. Results confirmed greater efficiency of players with visual impairment in attack and greater involvement of athletes with total blindness in defense, except in defense against penalty shots. It seems, again, that there could be specialization of goalball players. Players with visual impairment seem to be more active and efficient in offensive part of game, whereas players with total blindness specialize in defense.

There were no analyses of game performance in goalball available to the authors. Consequently, the authors used analyses from previous literature regarding assessment of game performance and set up game efficiency sheets for each player. Molik et al. (2009) and Vanlandewijck et al. (1995, 2003, 2004) analyzed game performance of wheelchair basketball athletes related to their classification levels. Another studies evaluated game performance of wheelchair rugby players (Morgulec-Adamowicz et al., 2010; Sarro et al., 2010; Sporner et al., 2009). Two studies were focused on performance of athletes with disabilities in ice sledge hockey (Kudláĉek et al., 2006; Molik et al., 2012).

Authors were also interested in which shooting techniques were efficient and typical for goalball players. In goalball, players prefer three different techniques: a spin throw (like a discus spin throw), a running throw, and a standing throw. During last year’s competitions, players employed spin throws more frequently due to the higher speed of the ball. However, teaching that technique is more time-consuming. Results confirmed that spin throws were used more frequently than running throws, but the success of the spin throws was lower. Coaches should note that the outcome of the spin throw is less successful. Maybe further development of training methods could be helpful for increasing shooting efficiency. Until those more effective training methods evolve, it seems that running shots should be preferred by goalball players.

Another analysis of the results allowed the description of the characteristics of elite goalball games. Analyses showed a rapid pace of the game with players defending and shooting the ball every minute. However, athletes with visual impairment (B2 & B3) take a higher number of shots in comparison to the number of defensive plays. Goalball players with total blindness are more active in defense than in offence.

The second aim of the study was to determine if game performance was related to anthropometric characteristics of elite male goalball players. The results did not show any significant relationships between anthropometric variables and game performance of elite players. Therefore, one would not expect body height, the arm span and length of the body in the defensive position in elite goalball players to influence effectiveness of play. Anthropometric variables do not differentiate players’ performance at the elite level. This is important information for coaches during the selection of individuals for elite goalball teams.

It is noted that there were only elite players analyzed in this research. Maybe further comparison of elite players to athletes representing levels below elite would demonstrate differences related to anthropometric variables.

There were some limitations and recommendations of this study. The number of athletes analyzed in the present research was relatively small. The authors decided to select elite athletes to reduce differences in performance levels between individuals. Additional investigation is needed to demonstrate performance of athletes according to shooting technique and the direction of shots. Also future studies should focus on team game performance to achieve clearer information regarding the keys for success in goalball.

## Practical Applications

Presented results confirmed that athletes with total blindness (B1) demonstrated higher activeness and efficiency in defensive part of the goalball game. However, athletes with visual impairment (B2 & B3) presented higher efficiency in offensive actions. Differences between both disability groups as well as athletes’ specialization should be useful for goalball coaching.

Additional analyses confirmed that body mass, height, the arm span and length of the body in the defensive position did not differentiate players’ performance at the elite level. That information is important for the selection process of top goalball athletes by coaches.

## Conclusion

Goalball players with visual impairment (B2 & B3) are more effective during the offensive phase of the game than those with total blindness (B1). The B1 athletes are more active on defense, except during the defense of penalty shots characterized.

## Figures and Tables

**Table 1:** Results of the U-Mann Whitney independent samples test between players with total blindness (B1) and players with visual impairment (B2/B3) (n = 44) for all the game indicators studied

	Totally Blind B1	Visually Impaired B2/B3		
			
*Game indicators*	Mean (*s*)	Mean (*s*)	Z	*p*
*Asum*	22.77 (14.60)	31.24 (13.66)	−1.57	0.21
*Aef*	2.50 (2.28)	4.28 (2.31)	−2.00	0.04*
*AG*	0.71 (.68)	1.24 (0.84)	−1.65	0.09
*AMsum*	22.06 (14.06)	30.05 (13.15)	−1.57	0.11
*AMS*	13.69 (8.74)	16.92 (7.85)	−1.03	0.31
*AMDO*	2.77 (1.84)	4.65 (2.96)	−1.82	0.07
*AMO*	2.16 (1.57)	3.32 (1.93)	−1.43	0.16
*AMLC*	3.22 (2.45)	4.88 (2.48)	−1.72	0.08
*AMB*	0.19 (.33)	0.22 (.25)	−0.74	0.78
*Dsum*	28.71 (5.89)	23.81 (5.19)	−2.07	0.03*
*Def*	97.10 (1.66)	95.69 (2.48)	−1.33	0.19
*DGsum*	27.89 (5.79)	22.83 (5.18)	−2.04	0.04*
*DGO*	4.02 (1.81)	2.91 (1.22)	−1.70	0.08
*DGL*	5.16 (3.14)	4.01 (2.33)	−0-08	0.09
*DGC*	16.29 (5.83)	13.99 (5.09)	−0.93	0.36
*DGS*	2.16 (1.18)	1.79 (1.02)	−0.69	0.51
*DGB*	0.05 (.04)	0.01 (.02)	−2.60	0.02*
*DN*	0.82 (.53)	0.97 (.46)	−0.83	0.42
*PDsum*	0.20 (.22)	0.89 (.81)	−2.98	0.01**
*PDef*	16.66 (32.48)	36.13 (36.20)	−1.65	0.13
*PDG*	0.06 (.11)	0.46 (.65)	−2.18	0.04*
*PDN*	0.14 (.19)	0.42 (.35)	−2.22	0.03*
*PAsum*	0.64 (.83)	0.55 (.62)	−0.02	0.98
*PAef*	23.15 (32.65)	36.19 (41.84)	−0.71	0.51
*PAG*	0.25 (.42)	0.34 (.45)	−0.53	0.54
*PAM*	0.39 (.45)	0.20 (.25)	−0.98	0.36
*FO*	0.23 (.26)	0.86 (.65)	−3.10	0.01**
*APtsum*	14.39 (14.20)	17.51 (17.28)	−0.96	0.34
*APtef*	1.49 (1.72)	2.18 (2.57)	−0.52	0.64
*APtG*	0.34 (.36)	0.62 (.76)	−0.62	0.57
*APtM*	14.04 (13.88)	16.88 (16.65)	−0.91	0.36
*ARtsum*	7.94 (12.73)	13.43 (16.21)	−1.01	0.31
*ARtef*	11.93 (29.80)	6.90 (12.05)	−0.56	0.61
*ARtG*	0.35 (.681)	0.61 (.82)	−1.24	0.25
*ARtM*	7.58 (12.14)	12.81 (15.66)	−1.13	0.27
*AStsum*	0.43 (.51)	0.30 (.36)	−0.73	0.48
*AStef*	4.54 (15.07)	0.00	−1.20	0.71
*AStG*	0.01 (.05)	0.00	−1.21	0.72
*AStM*	0.41 (.51)	0.30 (.36)	−0.58	0.57

* p < 0.05

** p < 0.01

B1 - visual acuity poorer than LogMAR 2.60; B2/B3 - visual acuity ranging from LogMAR 1 to 2.60 and/or visual field constricted to a diameter of less than 40 degrees. Asum = sum of attacks (shots); Aef = attack efficiency [%]; AG = good attack – goal; AMsum = sum of missed attacks; AMS = missed attack – a shot of the opponent immediately after defense; AMDO = missed attack - defense and ball out; AMO = missed attack – out; AMLC = missed attack - defense and lost contact with the ball; AMB = missed attack - defense and lost contact with the ball - ball back to the shooter; Dsum = sum of defense; Def = defense efficiency [%]; DGsum = sum of good defense; DGO = good defense – the ball out; DGL = good defense – a lost ball; DGC = good defense – the defender keeps contact with the ball; DGS = good defense – the second defender keeps contact with the ball; DGB = good defense - lost contact with the ball – the ball back to the opponent; DN = not good defense; PDsum = sum of penalty defences; PDef = penalty defense efficiency [%]; PDG = penalty good defense; PDN = penalty not good defense; PAsum = sum of penalty attacks; PAef = penalty efficiency attack; PAG = penalty attack good-goal; PAM = penalty attack missed; FO = foul; APtsum = sum of attacks spin throw technique; APtef = attack spin throw efficiency; APtG = attack spin throw good goal; APtM = attack spin throw missed; ARtsum = sum of attacks run technique; ARtef = attack run efficiency; ARtG = attack run good – goal; ARtM = attack run missed; AStsum = sum of attacks stand; AStef = attack stand efficiency; AStG = attack stand good – goal; AStM = attack stand missed.

**Table 2:** Descriptive results (mean ± SD) for anthropometric measurements of goalball players (n = 27) by the degree of visual impairment

	Totally Blind B1	Visually Impaired B2/B3
	
Anthropometric measurements	Mean (s)	Mean (s)
Body height [cm]	179.6 (38.47)	179.56 (5.48)
Body mass [kg]	83.28 (11.01)	85.07 (13.92)
Arm span [cm]	184.09 (7.31)	184.24 (9.09)
Length of the body in the defensive position [cm]	238.72 (12.85)	239.87 (8.67)

B1 - visual acuity poorer than LogMAR 2.60; B2/B3 - visual acuity ranging from LogMAR 1 to 2.60 and/or visual field constricted to a diameter of less than 40 degrees

**Table 3:** Results of the linear regression models predicting the influence of anthropometric variables (independent variables) on the game indicators (n = 27)

	Linear regression models						
	
Dependent variable	Aef	Def	PDef	FO	APtef	ARtef	AStef
Body height	−0.03 (0.14)	0.04 (0.13)	1.74 (1.81)	−0.01 (0.04)	−0.03 (0.13)	−0.86 (1.19)	0.53 (0.55)
Arm span	−0.12 (0.13)	−0.07 (0.12)	2.55 (1.63)	0.02 (0.03)	−0.17 (0.11)	−0.03 (1.06)	−0.40 (0.49)
Length of the body in the defensive position	−0.11 (0.11)	0.05 (0.11)	−1.61 (1.44)	0.01 (0.03)	0.16 (0.10)	0.90 (0.94)	−0.07 (0.44)
Intercept	3.96 (13.23)	90.62 (12.52)	−368.4 (171.8)	−2.20 (3.30)	1.16 (11.85)	−45.53 (112.4)	−37.13 (52.19)
R2	0.05	0.02	0.25	0.07	0.12	0.06	0.06
Number of observations	27	27	27	27	27	27	27

Standard error in parenthesis. Dependent variables: Aef = attack efficiency, Def = defense efficiency, PDef = penalty defense efficiency, FO = foul, APtef = attack spin throw efficiency, ARtef = attack run efficiency, AStef = attack stand efficiency
